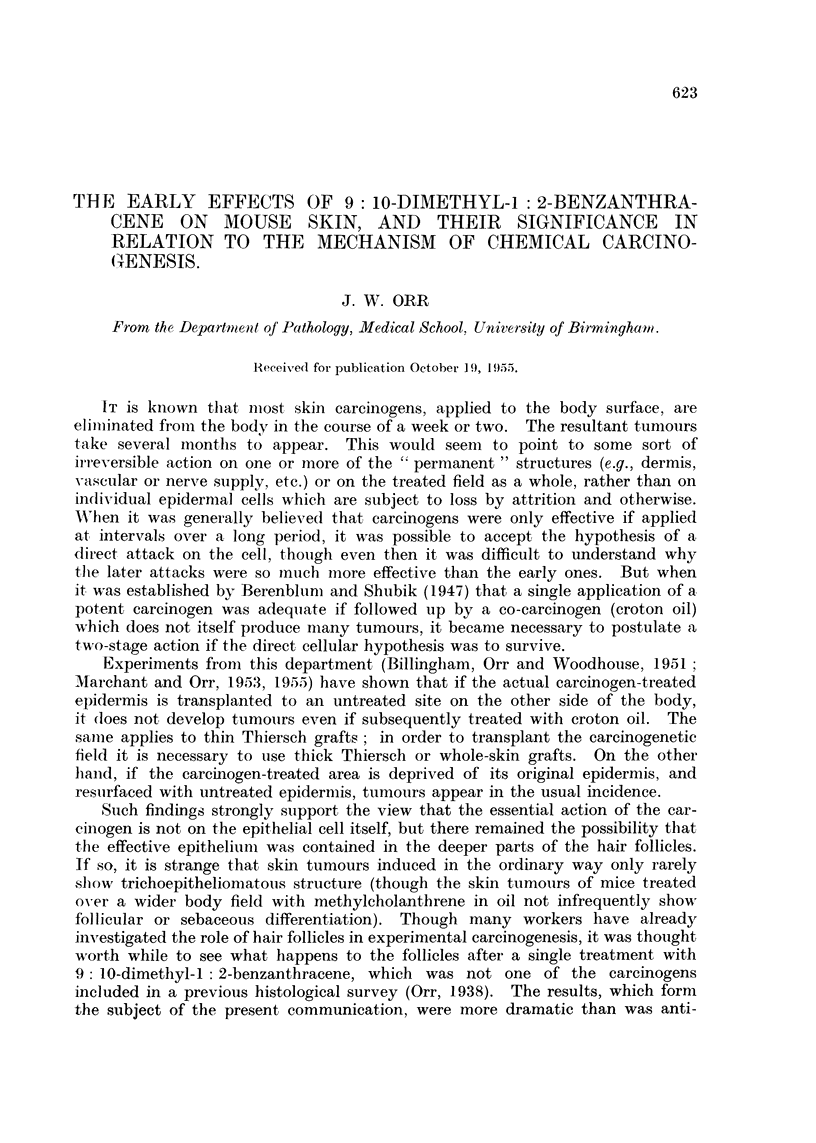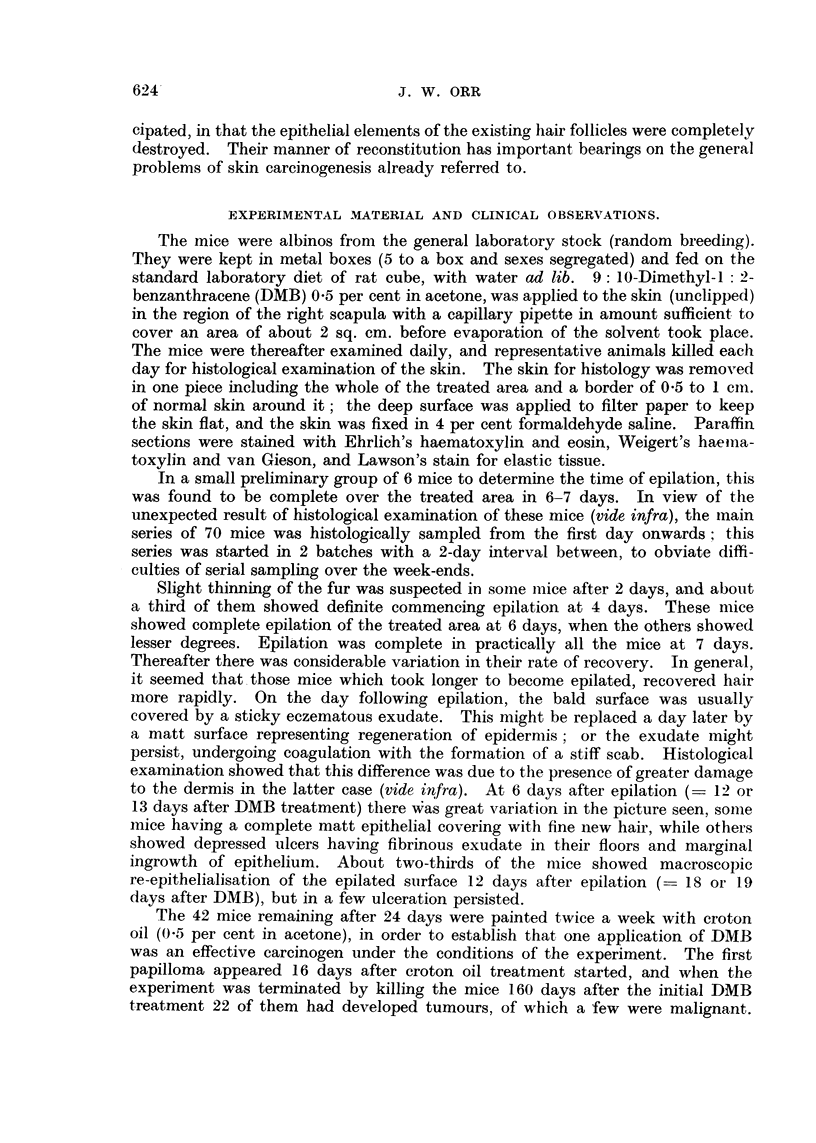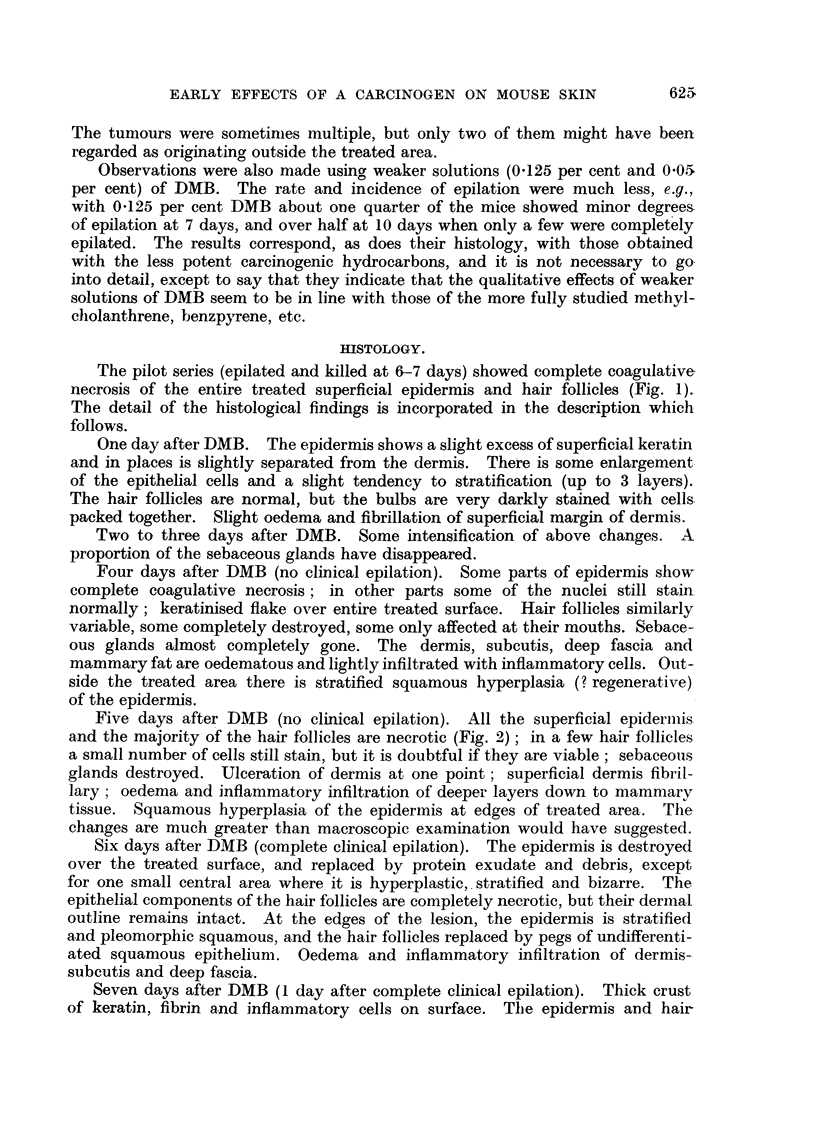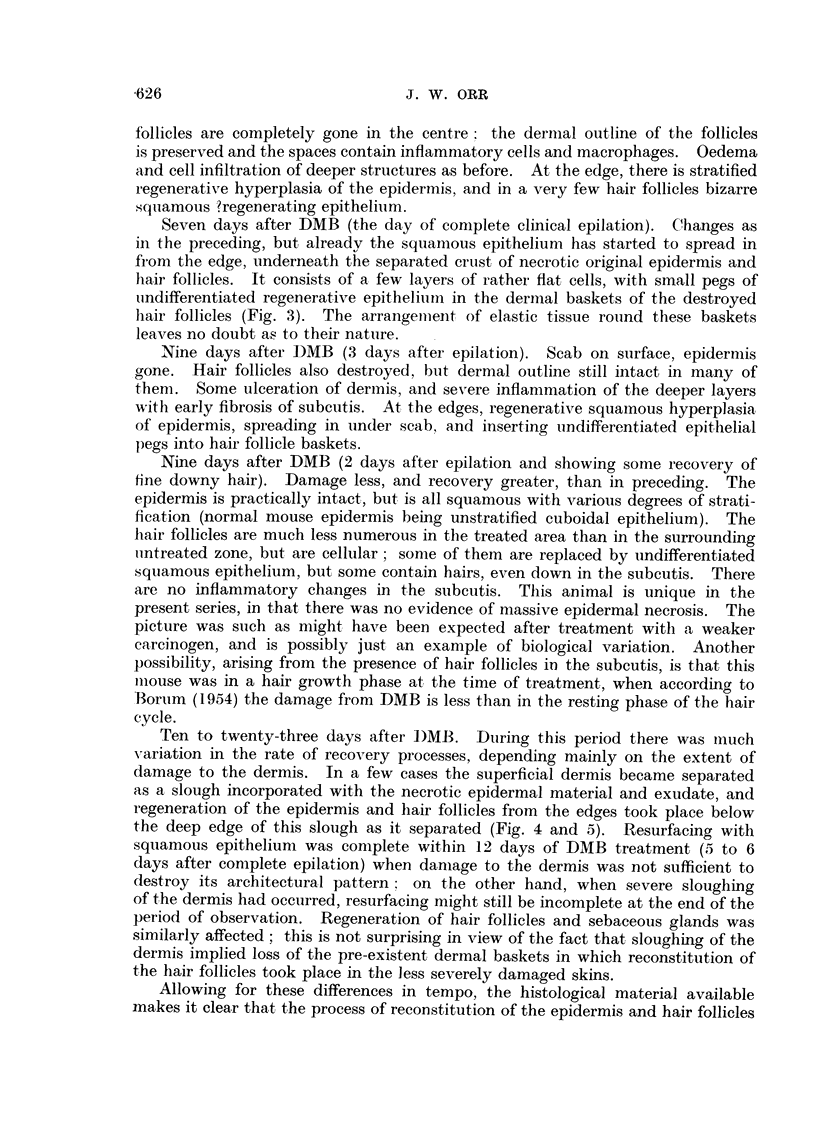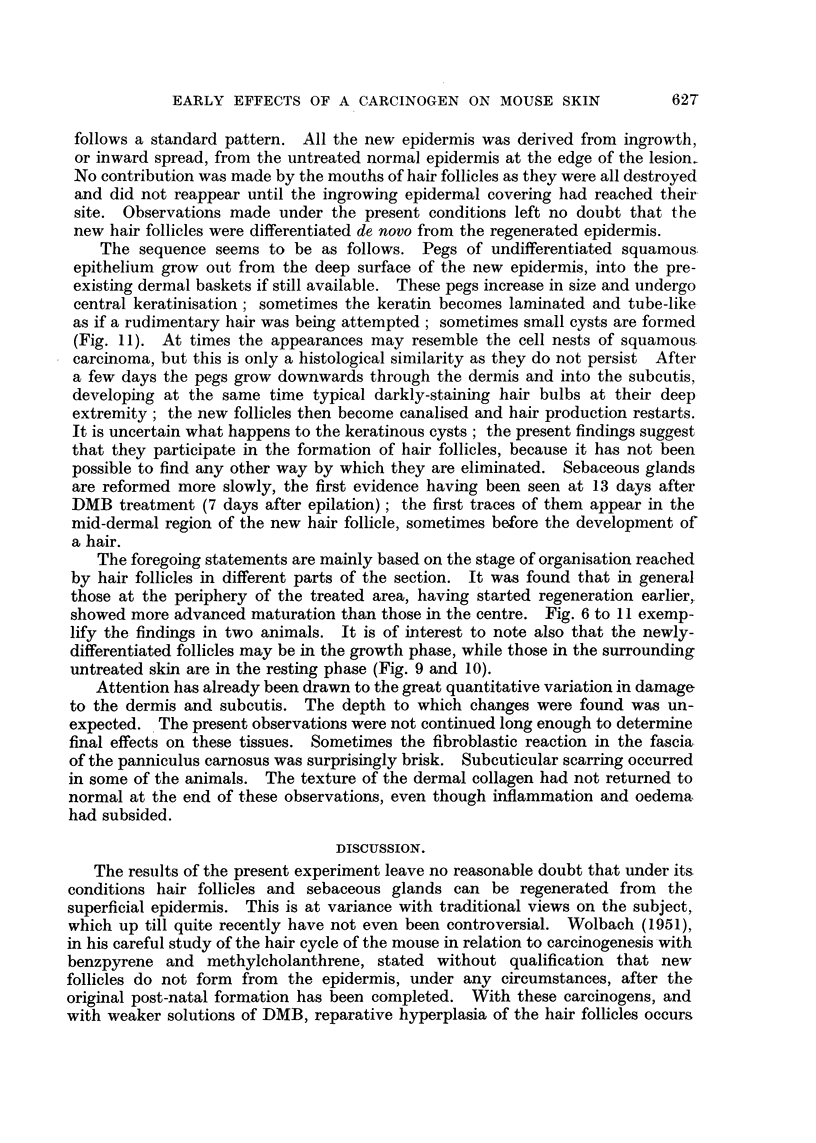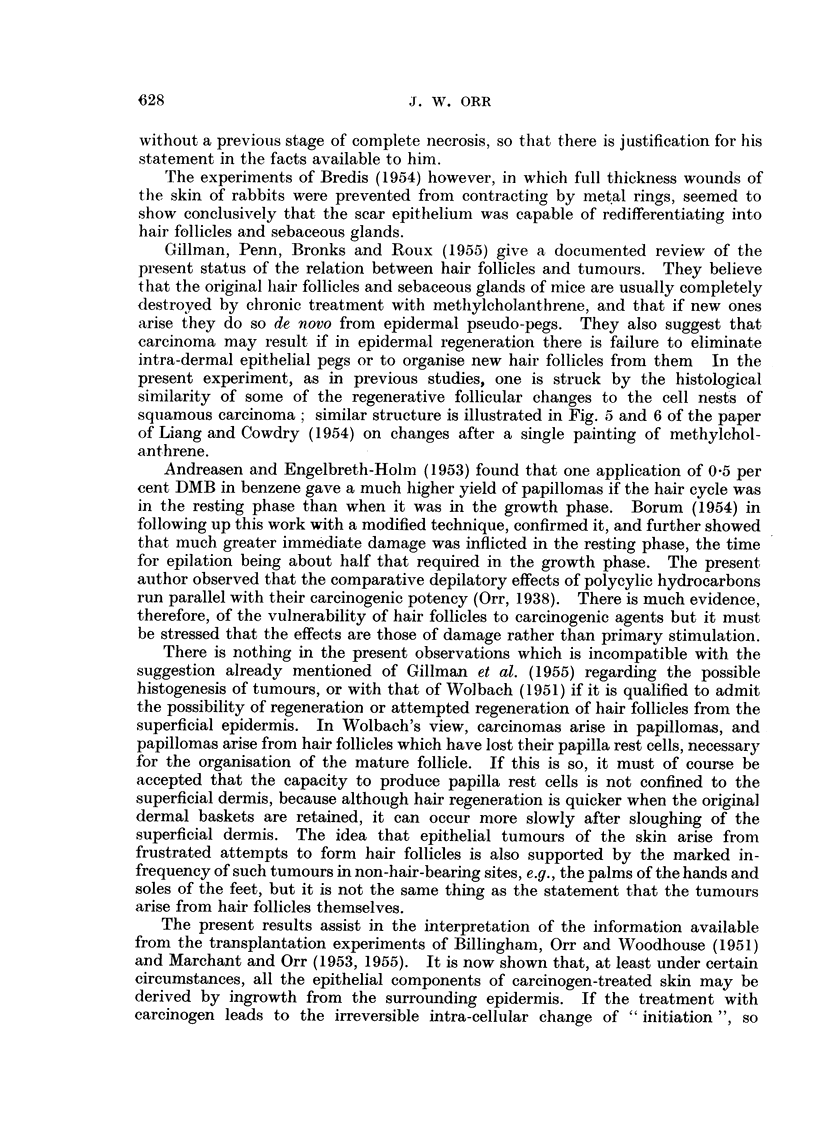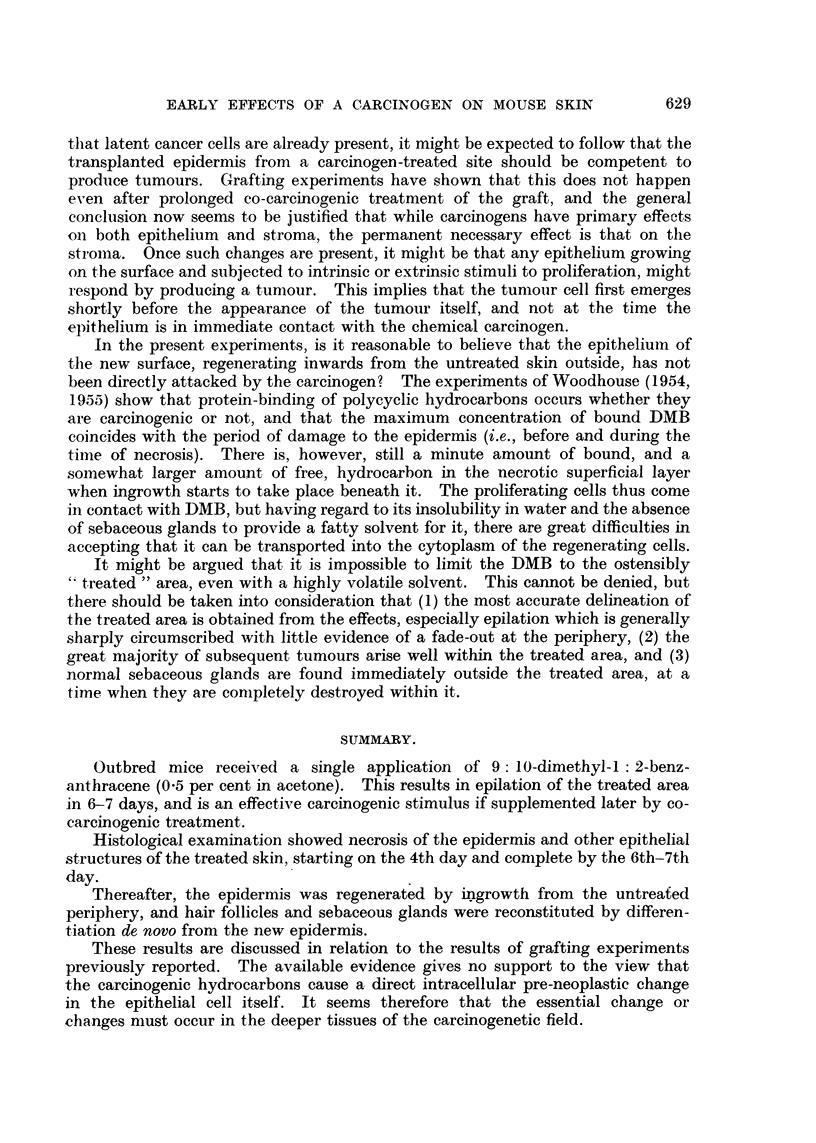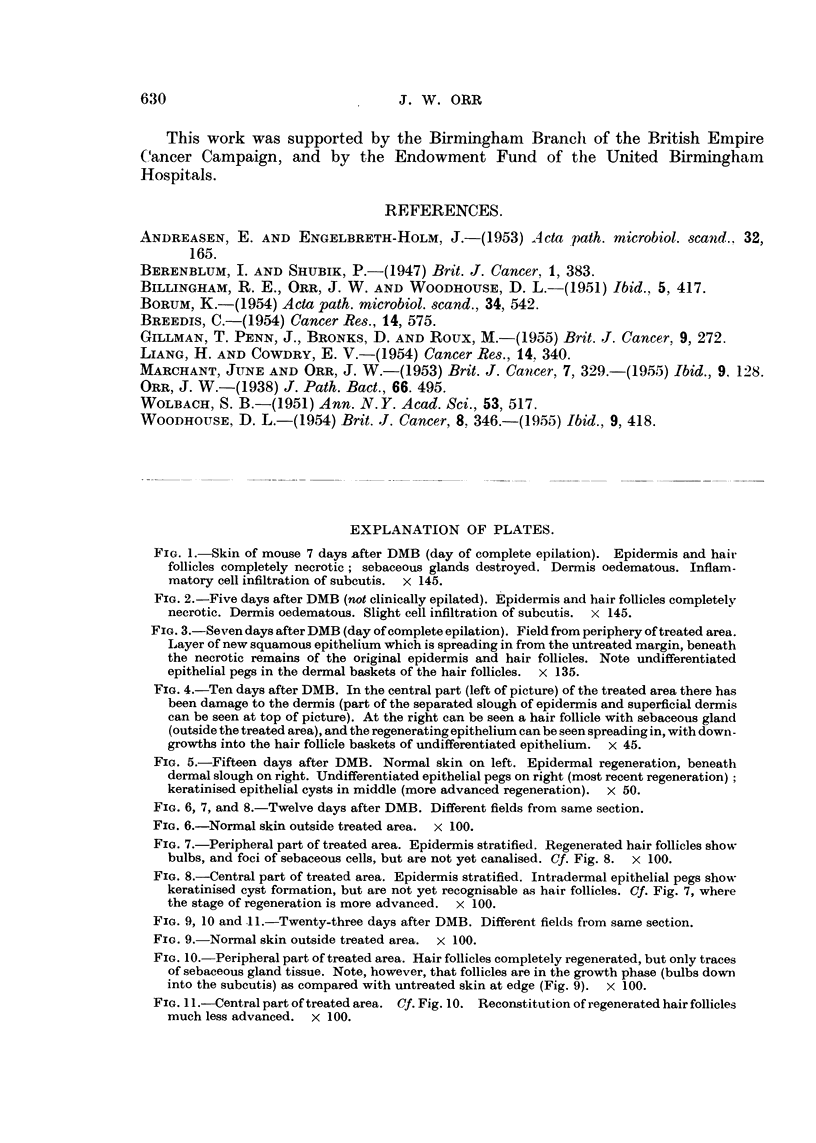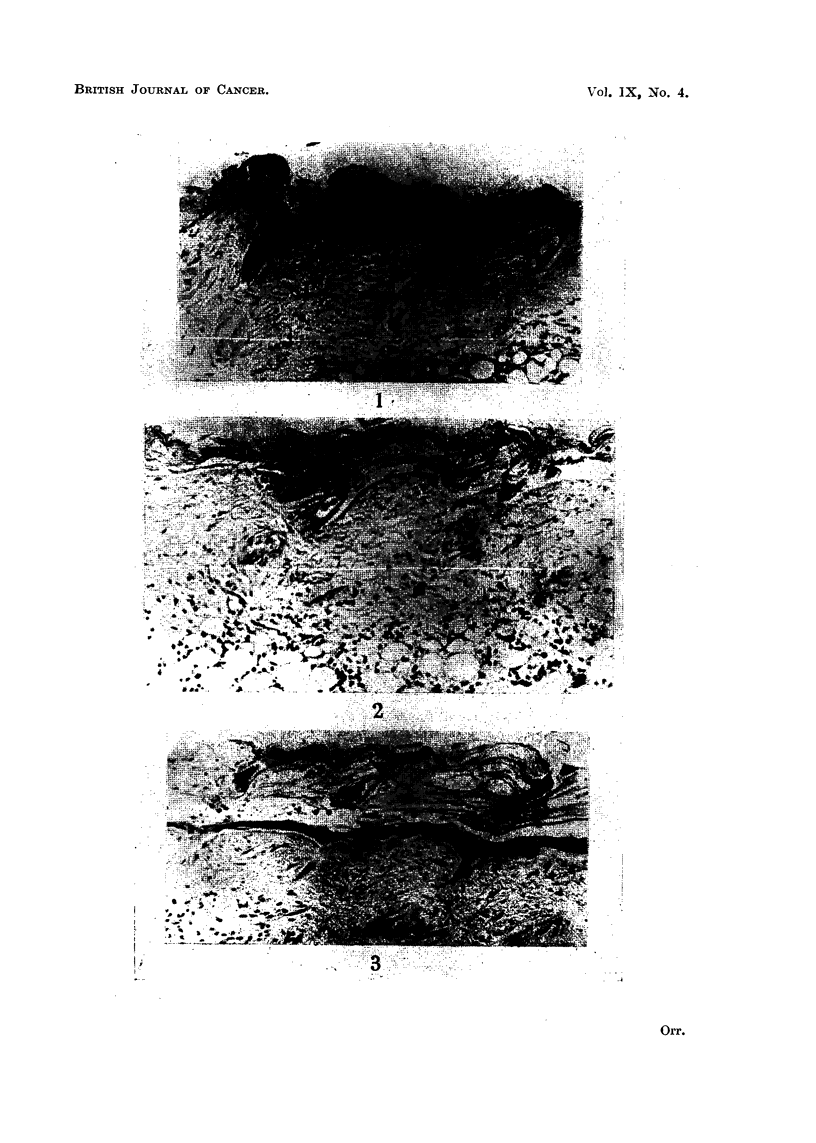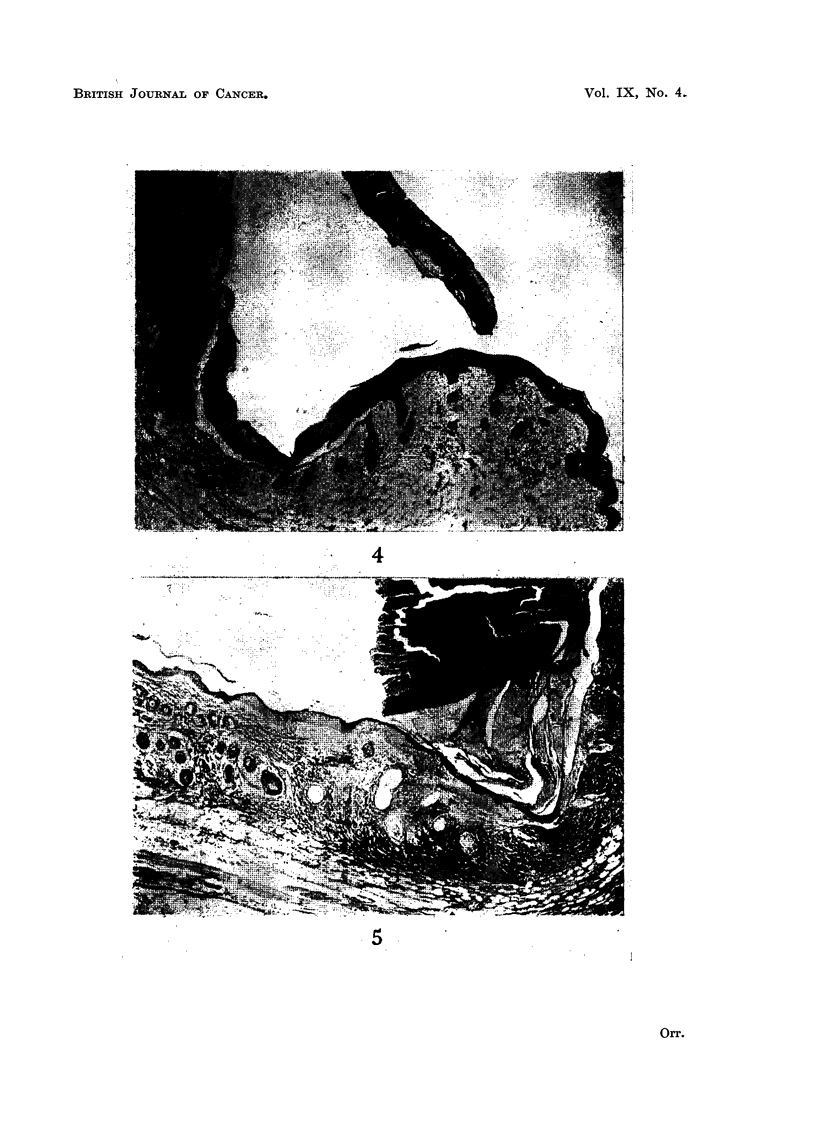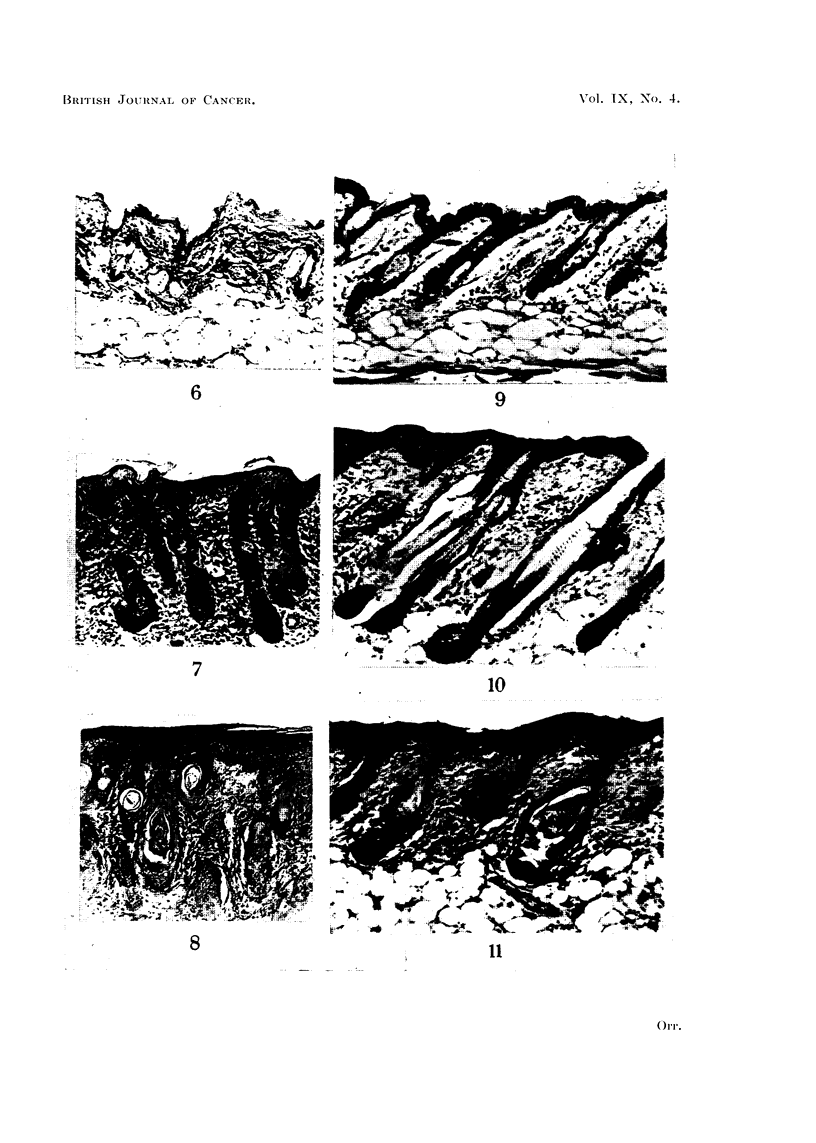# The Early Effects of 9: 10-Dimethyl-1:2-Benzanthracene on Mouse Skin, and their Significance in Relation to the Mechanism of Chemical Carcinogenesis

**DOI:** 10.1038/bjc.1955.66

**Published:** 1955-12

**Authors:** J. W. Orr

## Abstract

**Images:**


### THE EARLY EFFECTS OF 9 : 10-DIMETHYL-1 : 2-BENZANTHRACENE ON MOUSE SKIN, AND THEIR SIGNIFICANCE IN RELATION TO THE MECHANISM OF CHEMICAL CARCINOGENESIS.

J. W. ORR

*From the Department of Pathology, Medical School, University of Birmingham.*

Received for publication October 19, 1955.

IT is known that most skin carcinogens, applied to the body surface, are eliminated from the body in the course of a week or two. The resultant tumours take several months to appear. This would seem to point to some sort of irreversible action on one or more of the " permanent " structures (*e.g.*, dermis, vascular or nerve supply, etc.) or on the treated field as a whole, rather than on individual epidermal cells which are subject to loss by attrition and otherwise. When it was generally believed that carcinogens were only effective if applied at intervals over a long period, it was possible to accept the hypothesis of a direct attack on the cell, though even then it was difficult to understand why the later attacks were so much more effective than the early ones. But when it was established by Berenblum and Shubik (1947) that a single application of a potent carcinogen was adequate if followed up by a co-carcinogen (croton oil) which does not itself produce many tumours, it became necessary to postulate a two-stage action if the direct cellular hypothesis was to survive.

Experiments from this department (Billingham, Orr and Woodhouse, 1951 ; Marchant and Orr, 1953, 1955) have shown that if the actual carcinogen-treated epidermis is transplanted to an untreated site on the other side of the body, it does not develop tumours even if subsequently treated with croton oil. The same applies to thin Thiersch grafts ; in order to transplant the carcinogenetic field it is necessary to use thick Thiersch or whole-skin grafts. On the other hand, if the carcinogen-treated area is deprived of its original epidermis, and resurfaced with untreated epidermis, tumours appear in the usual incidence.

Such findings strongly support the view that the essential action of the carcinogen is not on the epithelial cell itself, but there remained the possibility that the effective epithelium was contained in the deeper parts of the hair follicles. If so, it is strange that skin tumours induced in the ordinary way only rarely show trichoepitheliomatous structure (though the skin tumours of mice treated over a wider body field with methylcholanthrene in oil not infrequently show follicular or sebaceous differentiation). Though many workers have already investigated the role of hair follicles in experimental carcinogenesis, it was thought worth while to see what happens to the follicles after a single treatment with 9 : 10-dimethyl-1 : 2-benzanthracene, which was not one of the carcinogens included in a previous histological survey (Orr, 1938). The results, which form the subject of the present communication, were more dramatic than was anticipated, in that the epithelial elements of the existing hair follicles were completely destroyed. Their manner of reconstitution has important bearings on the general problems of skin carcinogenesis already referred to.

### EXPERIMENTAL MATERIAL AND CLINICAL OBSERVATIONS.

The mice were albinos from the general laboratory stock (random breeding). They were kept in metal boxes (5 to a box and sexes segregated) and fed on the standard laboratory diet of rat cube, with water *ad lib.* 9 : 10-Dimethyl-1 : 2-benzanthracene (DMB) 0·5 per cent in acetone, was applied to the skin (unclipped) in the region of the right scapula with a capillary pipette in amount sufficient to cover an area of about 2 sq. cm. before evaporation of the solvent took place. The mice were thereafter examined daily, and representative animals killed each day for histological examination of the skin. The skin for histology was removed in one piece including the whole of the treated area and a border of 0·5 to 1 cm. of normal skin around it ; the deep surface was applied to filter paper to keep the skin flat, and the skin was fixed in 4 per cent formaldehyde saline. Paraffin sections were stained with Ehrlich's haematoxylin and eosin, Weigert's haematoxylin and van Gieson, and Lawson's stain for elastic tissue.

In a small preliminary group of 6 mice to determine the time of epilation, this was found to be complete over the treated area in 6–7 days. In view of the unexpected result of histological examination of these mice (*vide infra*), the main series of 70 mice was histologically sampled from the first day onwards ; this series was started in 2 batches with a 2-day interval between, to obviate difficulties of serial sampling over the week-ends.

Slight thinning of the fur was suspected in some mice after 2 days, and about a third of them showed definite commencing epilation at 4 days. These mice showed complete epilation of the treated area at 6 days, when the others showed lesser degrees. Epilation was complete in practically all the mice at 7 days. Thereafter there was considerable variation in their rate of recovery. In general, it seemed that those mice which took longer to become epilated, recovered hair more rapidly. On the day following epilation, the bald surface was usually covered by a sticky eczematous exudate. This might be replaced a day later by a matt surface representing regeneration of epidermis ; or the exudate might persist, undergoing coagulation with the formation of a stiff scab. Histological examination showed that this difference was due to the presence of greater damage to the dermis in the latter case (*vide infra*). At 6 days after epilation (= 12 or 13 days after DMB treatment) there was great variation in the picture seen, some mice having a complete matt epithelial covering with fine new hair, while others showed depressed ulcers having fibrinous exudate in their floors and marginal ingrowth of epithelium. About two-thirds of the mice showed macroscopic re-epithelialisation of the epilated surface 12 days after epilation (= 18 or 19 days after DMB), but in a few ulceration persisted.

The 42 mice remaining after 24 days were painted twice a week with croton oil (0·5 per cent in acetone), in order to establish that one application of DMB was an effective carcinogen under the conditions of the experiment. The first papilloma appeared 16 days after croton oil treatment started, and when the experiment was terminated by killing the mice 160 days after the initial DMB treatment 22 of them had developed tumours, of which a few were malignant.

The tumours were sometimes multiple, but only two of them might have been regarded as originating outside the treated area.

Observations were also made using weaker solutions (0·125 per cent and 0·05 per cent) of DMB. The rate and incidence of epilation were much less, *e.g.*, with 0·125 per cent DMB about one quarter of the mice showed minor degrees of epilation at 7 days, and over half at 10 days when only a few were completely epilated. The results correspond, as does their histology, with those obtained with the less potent carcinogenic hydrocarbons, and it is not necessary to go into detail, except to say that they indicate that the qualitative effects of weaker solutions of DMB seem to be in line with those of the more fully studied methylcholanthrene, benzpyrene, etc.

### HISTOLOGY.

The pilot series (epilated and killed at 6–7 days) showed complete coagulative necrosis of the entire treated superficial epidermis and hair follicles (Fig. 1). The detail of the histological findings is incorporated in the description which follows.

One day after DMB. The epidermis shows a slight excess of superficial keratin and in places is slightly separated from the dermis. There is some enlargement of the epithelial cells and a slight tendency to stratification (up to 3 layers). The hair follicles are normal, but the bulbs are very darkly stained with cells packed together. Slight oedema and fibrillation of superficial margin of dermis.

Two to three days after DMB. Some intensification of above changes. A proportion of the sebaceous glands have disappeared.

Four days after DMB (no clinical epilation). Some parts of epidermis show complete coagulative necrosis ; in other parts some of the nuclei still stain normally ; keratinised flake over entire treated surface. Hair follicles similarly variable, some completely destroyed, some only affected at their mouths. Sebaceous glands almost completely gone. The dermis, subcutis, deep fascia and mammary fat are oedematous and lightly infiltrated with inflammatory cells. Outside the treated area there is stratified squamous hyperplasia (? regenerative) of the epidermis.

Five days after DMB (no clinical epilation). All the superficial epidermis and the majority of the hair follicles are necrotic (Fig. 2) ; in a few hair follicles a small number of cells still stain, but it is doubtful if they are viable ; sebaceous glands destroyed. Ulceration of dermis at one point ; superficial dermis fibrillary ; oedema and inflammatory infiltration of deeper layers down to mammary tissue. Squamous hyperplasia of the epidermis at edges of treated area. The changes are much greater than macroscopic examination would have suggested.

Six days after DMB (complete clinical epilation). The epidermis is destroyed over the treated surface, and replaced by protein exudate and debris, except for one small central area where it is hyperplastic, stratified and bizarre. The epithelial components of the hair follicles are completely necrotic, but their dermal outline remains intact. At the edges of the lesion, the epidermis is stratified and pleomorphic squamous, and the hair follicles replaced by pegs of undifferentiated squamous epithelium. Oedema and inflammatory infiltration of dermis-subcutis and deep fascia.

Seven days after DMB (1 day after complete clinical epilation). Thick crust of keratin, fibrin and inflammatory cells on surface. The epidermis and hair follicles are completely gone in the centre: the dermal outline of the follicles is preserved and the spaces contain inflammatory cells and macrophages. Oedema and cell infiltration of deeper structures as before. At the edge, there is stratified regenerative hyperplasia of the epidermis, and in a very few hair follicles bizarre squamous ?regenerating epithelium.

Seven days after DMB (the day of complete clinical epilation). Changes as in the preceding, but already the squamous epithelium has started to spread in from the edge, underneath the separated crust of necrotic original epidermis and hair follicles. It consists of a few layers of rather flat cells, with small pegs of undifferentiated regenerative epithelium in the dermal baskets of the destroyed hair follicles (Fig. 3). The arrangement of elastic tissue round these baskets leaves no doubt as to their nature.

Nine days after DMB (3 days after epilation). Scab on surface, epidermis gone. Hair follicles also destroyed, but dermal outline still intact in many of them. Some ulceration of dermis, and severe inflammation of the deeper layers with early fibrosis of subcutis. At the edges, regenerative squamous hyperplasia of epidermis, spreading in under scab, and inserting undifferentiated epithelial pegs into hair follicle baskets.

Nine days after DMB (2 days after epilation and showing some recovery of fine downy hair). Damage less, and recovery greater, than in preceding. The epidermis is practically intact, but is all squamous with various degrees of stratification (normal mouse epidermis being unstratified cuboidal epithelium). The hair follicles are much less numerous in the treated area than in the surrounding untreated zone, but are cellular; some of them are replaced by undifferentiated squamous epithelium, but some contain hairs, even down in the subcutis. There are no inflammatory changes in the subcutis. This animal is unique in the present series, in that there was no evidence of massive epidermal necrosis. The picture was such as might have been expected after treatment with a weaker carcinogen, and is possibly just an example of biological variation. Another possibility, arising from the presence of hair follicles in the subcutis, is that this mouse was in a hair growth phase at the time of treatment, when according to Borum (1954) the damage from DMB is less than in the resting phase of the hair cycle.

Ten to twenty-three days after DMB. During this period there was much variation in the rate of recovery processes, depending mainly on the extent of damage to the dermis. In a few cases the superficial dermis became separated as a slough incorporated with the necrotic epidermal material and exudate, and regeneration of the epidermis and hair follicles from the edges took place below the deep edge of this slough as it separated (Fig. 4 and 5). Resurfacing with squamous epithelium was complete within 12 days of DMB treatment (5 to 6 days after complete epilation) when damage to the dermis was not sufficient to destroy its architectural pattern; on the other hand, when severe sloughing of the dermis had occurred, resurfacing might still be incomplete at the end of the period of observation. Regeneration of hair follicles and sebaceous glands was similarly affected; this is not surprising in view of the fact that sloughing of the dermis implied loss of the pre-existent dermal baskets in which reconstitution of the hair follicles took place in the less severely damaged skins.

Allowing for these differences in tempo, the histological material available makes it clear that the process of reconstitution of the epidermis and hair follicles follows a standard pattern. All the new epidermis was derived from ingrowth, or inward spread, from the untreated normal epidermis at the edge of the lesion. No contribution was made by the mouths of hair follicles as they were all destroyed and did not reappear until the ingrowing epidermal covering had reached their site. Observations made under the present conditions left no doubt that the new hair follicles were differentiated *de novo* from the regenerated epidermis.

The sequence seems to be as follows. Pegs of undifferentiated squamous epithelium grow out from the deep surface of the new epidermis, into the pre-existing dermal baskets if still available. These pegs increase in size and undergo central keratinisation ; sometimes the keratin becomes laminated and tube-like as if a rudimentary hair was being attempted ; sometimes small cysts are formed (Fig. 11). At times the appearances may resemble the cell nests of squamous carcinoma, but this is only a histological similarity as they do not persist After a few days the pegs grow downwards through the dermis and into the subcutis, developing at the same time typical darkly-staining hair bulbs at their deep extremity ; the new follicles then become canalised and hair production restarts. It is uncertain what happens to the keratinous cysts ; the present findings suggest that they participate in the formation of hair follicles, because it has not been possible to find any other way by which they are eliminated. Sebaceous glands are reformed more slowly, the first evidence having been seen at 13 days after DMB treatment (7 days after epilation) ; the first traces of them appear in the mid-dermal region of the new hair follicle, sometimes before the development of a hair.

The foregoing statements are mainly based on the stage of organisation reached by hair follicles in different parts of the section. It was found that in general those at the periphery of the treated area, having started regeneration earlier, showed more advanced maturation than those in the centre. Fig. 6 to 11 exemplify the findings in two animals. It is of interest to note also that the newly-differentiated follicles may be in the growth phase, while those in the surrounding untreated skin are in the resting phase (Fig. 9 and 10).

Attention has already been drawn to the great quantitative variation in damage to the dermis and subcutis. The depth to which changes were found was unexpected. The present observations were not continued long enough to determine final effects on these tissues. Sometimes the fibroblastic reaction in the fascia of the panniculus carnosus was surprisingly brisk. Subcuticular scarring occurred in some of the animals. The texture of the dermal collagen had not returned to normal at the end of these observations, even though inflammation and oedema had subsided.

### DISCUSSION.

The results of the present experiment leave no reasonable doubt that under its conditions hair follicles and sebaceous glands can be regenerated from the superficial epidermis. This is at variance with traditional views on the subject, which up till quite recently have not even been controversial. Wolbach (1951), in his careful study of the hair cycle of the mouse in relation to carcinogenesis with benzpyrene and methylcholanthrene, stated without qualification that new follicles do not form from the epidermis, under any circumstances, after the original post-natal formation has been completed. With these carcinogens, and with weaker solutions of DMB, reparative hyperplasia of the hair follicles occurs without a previous stage of complete necrosis, so that there is justification for his statement in the facts available to him.

The experiments of Bredis (1954) however, in which full thickness wounds of the skin of rabbits were prevented from contracting by metal rings, seemed to show conclusively that the scar epithelium was capable of redifferentiating into hair follicles and sebaceous glands.

Gillman, Penn, Bronks and Roux (1955) give a documented review of the present status of the relation between hair follicles and tumours. They believe that the original hair follicles and sebaceous glands of mice are usually completely destroyed by chronic treatment with methylcholanthrene, and that if new ones arise they do so *de novo* from epidermal pseudo-pegs. They also suggest that carcinoma may result if in epidermal regeneration there is failure to eliminate intra-dermal epithelial pegs or to organise new hair follicles from them In the present experiment, as in previous studies, one is struck by the histological similarity of some of the regenerative follicular changes to the cell nests of squamous carcinoma ; similar structure is illustrated in Fig. 5 and 6 of the paper of Liang and Cowdry (1954) on changes after a single painting of methylcholanthrene.

Andreasen and Engelbreth-Holm (1953) found that one application of 0·5 per cent DMB in benzene gave a much higher yield of papillomas if the hair cycle was in the resting phase than when it was in the growth phase. Borum (1954) in following up this work with a modified technique, confirmed it, and further showed that much greater immediate damage was inflicted in the resting phase, the time for epilation being about half that required in the growth phase. The present author observed that the comparative depilatory effects of polycylic hydrocarbons run parallel with their carcinogenic potency (Orr, 1938). There is much evidence, therefore, of the vulnerability of hair follicles to carcinogenic agents but it must be stressed that the effects are those of damage rather than primary stimulation.

There is nothing in the present observations which is incompatible with the suggestion already mentioned of Gillman *et al.* (1955) regarding the possible histogenesis of tumours, or with that of Wolbach (1951) if it is qualified to admit the possibility of regeneration or attempted regeneration of hair follicles from the superficial epidermis. In Wolbach's view, carcinomas arise in papillomas, and papillomas arise from hair follicles which have lost their papilla rest cells, necessary for the organisation of the mature follicle. If this is so, it must of course be accepted that the capacity to produce papilla rest cells is not confined to the superficial dermis, because although hair regeneration is quicker when the original dermal baskets are retained, it can occur more slowly after sloughing of the superficial dermis. The idea that epithelial tumours of the skin arise from frustrated attempts to form hair follicles is also supported by the marked infrequency of such tumours in non-hair-bearing sites, *e.g.*, the palms of the hands and soles of the feet, but it is not the same thing as the statement that the tumours arise from hair follicles themselves.

The present results assist in the interpretation of the information available from the transplantation experiments of Billingham, Orr and Woodhouse (1951) and Marchant and Orr (1953, 1955). It is now shown that, at least under certain circumstances, all the epithelial components of carcinogen-treated skin may be derived by ingrowth from the surrounding epidermis. If the treatment with carcinogen leads to the irreversible intra-cellular change of " initiation ", so that latent cancer cells are already present, it might be expected to follow that the transplanted epidermis from a carcinogen-treated site should be competent to produce tumours. Grafting experiments have shown that this does not happen even after prolonged co-carcinogenic treatment of the graft, and the general conclusion now seems to be justified that while carcinogens have primary effects on both epithelium and stroma, the permanent necessary effect is that on the stroma. Once such changes are present, it might be that any epithelium growing on the surface and subjected to intrinsic or extrinsic stimuli to proliferation, might respond by producing a tumour. This implies that the tumour cell first emerges shortly before the appearance of the tumour itself, and not at the time the epithelium is in immediate contact with the chemical carcinogen.

In the present experiments, is it reasonable to believe that the epithelium of the new surface, regenerating inwards from the untreated skin outside, has not been directly attacked by the carcinogen? The experiments of Woodhouse (1954, 1955) show that protein-binding of polycyclic hydrocarbons occurs whether they are carcinogenic or not, and that the maximum concentration of bound DMB coincides with the period of damage to the epidermis (*i.e.*, before and during the time of necrosis). There is, however, still a minute amount of bound, and a somewhat larger amount of free, hydrocarbon in the necrotic superficial layer when ingrowth starts to take place beneath it. The proliferating cells thus come in contact with DMB, but having regard to its insolubility in water and the absence of sebaceous glands to provide a fatty solvent for it, there are great difficulties in accepting that it can be transported into the cytoplasm of the regenerating cells.

It might be argued that it is impossible to limit the DMB to the ostensibly " treated " area, even with a highly volatile solvent. This cannot be denied, but there should be taken into consideration that (1) the most accurate delineation of the treated area is obtained from the effects, especially epilation which is generally sharply circumscribed with little evidence of a fade-out at the periphery, (2) the great majority of subsequent tumours arise well within the treated area, and (3) normal sebaceous glands are found immediately outside the treated area, at a time when they are completely destroyed within it.

### SUMMARY.

Outbred mice received a single application of 9 : 10-dimethyl-1 : 2-benzanthracene (0·5 per cent in acetone). This results in epilation of the treated area in 6–7 days, and is an effective carcinogenic stimulus if supplemented later by co-carcinogenic treatment.

Histological examination showed necrosis of the epidermis and other epithelial structures of the treated skin, starting on the 4th day and complete by the 6th–7th day.

Thereafter, the epidermis was regenerated by ingrowth from the untreated periphery, and hair follicles and sebaceous glands were reconstituted by differentiation *de novo* from the new epidermis.

These results are discussed in relation to the results of grafting experiments previously reported. The available evidence gives no support to the view that the carcinogenic hydrocarbons cause a direct intracellular pre-neoplastic change in the epithelial cell itself. It seems therefore that the essential change or changes must occur in the deeper tissues of the carcinogenetic field.

This work was supported by the Birmingham Branch of the British Empire Cancer Campaign, and by the Endowment Fund of the United Birmingham Hospitals.

### REFERENCES.

ANDREASEN, E. AND ENGELBRETH-HOLM, J.—(1953) *Acta path. microbiol. scand.*, **32**, 165.
BERENBLUM, I. AND SHUBIK, P.—(1947) *Brit. J. Cancer*, **1**, 383.
BILLINGHAM, R. E., ORR, J. W. AND WOODHOUSE, D. L.—(1951) *Ibid.*, **5**, 417.
BORUM, K.—(1954) *Acta path. microbiol. scand.*, **34**, 542.
BREEDIS, C.—(1954) *Cancer Res.*, **14**, 575.
GILLMAN, T. PENN, J., BRONKS, D. AND ROUX, M.—(1955) *Brit. J. Cancer*, **9**, 272.
LIANG, H. AND COWDRY, E. V.—(1954) *Cancer Res.*, **14**, 340.
MARCHANT, JUNE AND ORR, J. W.—(1953) *Brit. J. Cancer*, **7**, 329.—(1955) *Ibid.*, **9**, 128.
ORR, J. W.—(1938) *J. Path. Bact.*, **66**. 495.
WOLBACH, S. B.—(1951) *Ann. N.Y. Acad. Sci.*, **53**, 517.
WOODHOUSE, D. L.—(1954) *Brit. J. Cancer*, **8**, 346.—(1955) *Ibid.*, **9**, 418.

### EXPLANATION OF PLATES.

FIG. 1.—Skin of mouse 7 days after DMB (day of complete epilation). Epidermis and hair follicles completely necrotic ; sebaceous glands destroyed. Dermis oedematous. Inflammatory cell infiltration of subcutis. × 145.

FIG. 2.—Five days after DMB (*not* clinically epilated). Epidermis and hair follicles completely necrotic. Dermis oedematous. Slight cell infiltration of subcutis. × 145.

FIG. 3.—Seven days after DMB (day of complete epilation). Field from periphery of treated area. Layer of new squamous epithelium which is spreading in from the untreated margin, beneath the necrotic remains of the original epidermis and hair follicles. Note undifferentiated epithelial pegs in the dermal baskets of the hair follicles. × 135.

FIG. 4.—Ten days after DMB. In the central part (left of picture) of the treated area there has been damage to the dermis (part of the separated slough of epidermis and superficial dermis can be seen at top of picture). At the right can be seen a hair follicle with sebaceous gland (outside the treated area), and the regenerating epithelium can be seen spreading in, with downgrowths into the hair follicle baskets of undifferentiated epithelium. × 45.

FIG. 5.—Fifteen days after DMB. Normal skin on left. Epidermal regeneration, beneath dermal slough on right. Undifferentiated epithelial pegs on right (most recent regeneration) ; keratinised epithelial cysts in middle (more advanced regeneration). × 50.

FIG. 6, 7, and 8.—Twelve days after DMB. Different fields from same section.

FIG. 6.—Normal skin outside treated area. × 100.

FIG. 7.—Peripheral part of treated area. Epidermis stratified. Regenerated hair follicles show bulbs, and foci of sebaceous cells, but are not yet canalised. *Cf.* Fig. 8. × 100.

FIG. 8.—Central part of treated area. Epidermis stratified. Intradermal epithelial pegs show keratinised cyst formation, but are not yet recognisable as hair follicles. *Cf.* Fig. 7, where the stage of regeneration is more advanced. × 100.

FIG. 9, 10 and 11.—Twenty-three days after DMB. Different fields from same section.

FIG. 9.—Normal skin outside treated area. × 100.

FIG. 10.—Peripheral part of treated area. Hair follicles completely regenerated, but only traces of sebaceous gland tissue. Note, however, that follicles are in the growth phase (bulbs down into the subcutis) as compared with untreated skin at edge (Fig. 9). × 100.

FIG. 11.—Central part of treated area. *Cf.* Fig. 10. Reconstitution of regenerated hair follicles much less advanced. × 100.